# Transcriptome Dynamics Provide Insight into the Mechanisms Underlying Cucumber Stomatal Movement Regulated by Blue Light (BL) and Drought Stress

**DOI:** 10.3390/ijms27093717

**Published:** 2026-04-22

**Authors:** Xinying Liu, Qiying Sun, Zheng Wang, Yaliang Xu, Xin Liu, Sujun Liu, Binbin Liu, Qingming Li

**Affiliations:** 1 Institute of Urban Agriculture, Chinese Academy of Agricultural Sciences, Chengdu 610213, China; liuxinying@caas.cn (X.L.); wangzheng@caas.cn (Z.W.); xuyaliang@caas.cn (Y.X.); lxinanhao@outlook.com (X.L.); 2 National Chengdu Agricultural Science and Technology Center, Chengdu 610213, China; sunqiying@caas.cn (Q.S.); liusujun213@163.com (S.L.); 3 College of Food and Biological Engineering, Chengdu University, Chengdu 610106, China; 4 Key Laboratory of Plant Factory Speed Breeding, Ministry of Agriculture and Rural Affairs, Chengdu 610213, China

**Keywords:** light quality, drought stress, photosynthetic parameters, stomatal movement, water/fluid transport, aquaporins, transcriptomics

## Abstract

Light and drought antagonistically regulate stomatal movement, yet the mechanisms for integrating these conflicting signals remain unclear. In this study, the stomatal aperture and photosynthetic parameters under red light (RL), blue light (BL), and white light in different water regimes were evaluated. Transcriptome analysis was conducted during a 0–6 h period of BL exposure, with or without drought, to explore the molecular mechanisms underlying BL and drought-mediated stomatal movement. Under non-drought conditions, BL significantly enhanced stomatal conductance, transpiration rate, and stomatal aperture. After drought stress, BL-treated seedlings exhibited the greatest reductions in these indicators. Transcriptomic analysis revealed that both BL-responsive genes and drought-responsive genes were significantly enriched in overlapping pathways related to plant hormone signal transduction, and biological processes of water/fluid transport. Among these, the aquaporin gene *CsPIP2;3* was identified as a core node in the crosstalk between BL and drought signals, and a potential key regulator of stomatal movement. Tissue-specific expression analysis showed its highest expression in mature leaves; GUS staining further confirmed its expression in guard cells and vascular bundles, while subcellular localization verified the plasma membrane localization of its encoded protein. The transcriptomic data provide novel insights into the mechanisms underlying stomatal movement regulated by BL and drought.

## 1. Introduction

The frequent occurrence of extreme weather events, including droughts, high temperatures, and other extreme weather, significantly restricts sustainable crop production [1,2]. Within this context, stomata, as the central structures regulating gas exchange and water balance between plants and the environment, serve as critical interfaces for plant responses to multifaceted climatic stressors [3,4]. This pivotal role manifests in stomatal movement, and specifically when environmental fluctuations induced by climate change occur, plants rely on dynamic stomatal aperture adjustments to adapt to new environmental demands [5].

The dynamic balance between water loss and CO_2_ uptake, regulated by stomatal movement, requires precise coordination between external signals (e.g., light, temperature, external humidity, and CO_2_ concentration) and internal signaling (e.g., abscisic acid (ABA), salicylic acid (SA), and jasmonic acid (JA)) [5,6]. Stomatal opening to light is driven by two distinct pathways: A red light (RL) pathway–stomatal opening exhibits saturation kinetics at high irradiance, primarily driven by photosynthetic activation in guard cell chloroplasts, which reduces intercellular CO_2_ concentration (*C*_i_) and subsequently promotes phosphorylation of plasma membrane H^+^-ATPase in guard cells, ultimately driving stomatal aperture expansion at the whole-leaf level [7]. In contrast, the guard cell-specific blue light (BL) pathway, which is characterized by low-fluence saturation kinetics, is triggered by phototropin (Phot1/Phot2) autophosphorylation [8]. The primary phosphorylation target of phototropins is Blue Light Signaling1 (BLUS1), a key component of the initial BL signaling complex that drives stomatal opening [9]. The phosphorylated BLUS1 kinase induces phosphorylation of the penultimate threonine residue in the plasma membrane H^+^-ATPase. Concurrently, the phosphorylated C-terminus of H^+^-ATPase in guard cells recruits 14-3-3 proteins, a family of highly conserved regulatory adapter proteins, that specifically bind to phosphorylated target proteins, thereby relieving C-terminal auto-inhibition. This dual modification triggers phosphorylation-mediated activation of guard cell H^+^-ATPase, resulting in plasma membrane hyperpolarization [10]. The generated electrochemical gradient drives K^+^ uptake through voltage-gated inward-rectifying K^+^ channels and anion accumulation (through Cl^−^ channels and aluminum-activated malate transporter 9 (ALMT9) malate transporters), collectively enhancing water uptake and turgor pressure in guard cells, thereby promoting stomatal aperture expansion [11,12]. Although the individual pathways for light-induced stomatal opening are well-characterized, most studies have focused on single light qualities under non-stress conditions. Therefore, how different light qualities, particularly RL and BL, modulate stomatal movement under drought stress, a commonly co-occurring environmental factor, remains largely unexplored.

Drought is a key environmental cue inducing stomatal closure [13]. Under drought stress, ABA regulates stomatal movement through two synergistic and partially parallel pathways: the direct guard cell-autonomous pathway and the indirect non-cell-autonomous pathway, which acts on other leaf cell types, including mesophyll cells (MCs) and bundle sheath cells (BSCs). This multicellular coordinated regulatory network ultimately drives stomatal closure to reduce transpiration water loss and maintain plant water balance [14,15]. In recent years, studies on the regulation of stomatal closure by ABA have largely focused on guard cells themselves. Under low and subthreshold concentrations of ABA, protein phosphatase 2C (PP2C) ABA-insensitive 1 (ABI1) and 2 (ABI2), negative regulatory factors of ABA signaling, inhibit positive regulators of stomatal closure such as the protein kinase Open Stomata 1 (OST1), or the calcium-dependent protein kinases (CPKs), keeping the downstream transcription factors and guard cell anion channels in an inactive state [16,17]. Under drought stress, ABA rapidly accumulates and binds to its receptor complex, forming the PYR/PYL/RCAR-ABA complex. Subsequently, this complex interacts with PP2C phosphatases (including ABI1 and ABI2), inhibiting their activity and thereby activating downstream kinases such as OST1, CPKs, and GHR1 [18,19]. The active kinases then activate slow (S)- and rapid (R)-type anion channels, which in turn cause plasma membrane depolarization and promote the efflux of Cl^−^, malate, and NO_3_^−^ [13,19]. The consequent ion efflux lowers cellular osmotic potential and triggers rapid water efflux from guard cells. This ultimately reduces the turgor pressure of guard cells, driving cellular volume reduction and stomatal closure [13,20]. ABA can also promote stomatal closure via an indirect hydraulic effect, independent of the guard cell-autonomous signaling pathway described above, by reducing water permeability in leaf vascular tissues [21]. Specifically, ABA may act on cells surrounding leaf vascular bundles, such as xylem BSCs, and modulate water transport routes and leaf hydraulic conductivity (K_leaf_) by regulating the activity or abundance of aquaporins (AQPs) in the plasma membranes of these cells, ultimately leading to stomatal closure [14]. Additionally, the regulation of water transport in leaves alters cellular turgor pressure. The resulting reduction in turgor of mesophyll and epidermal cells drives cell contraction, and the associated mechanical forces propagate to adjacent guard cells through intercellular physical connections within the leaf tissue [22]. These turgor-driven changes in cell volume and cell wall tension generate a mechanical signal, which is perceived by mechanosensitive proteins (e.g., mechanosensitive ion channels) on the guard cell plasma membrane, thereby triggering or amplifying the intracellular ABA signaling cascade in guard cells and ultimately driving stomatal closure [23].

Given that light and drought often act as antagonistic signals for stomatal movement, a crucial question arises: how do plants integrate these conflicting cues to optimize their physiological responses? Investigating this question can reveal how plants integrate multiple environmental signals to optimize survival strategies, elucidating the molecular mechanisms underlying stomatal movement. Cucumber is an economically important crop often cultivated in controlled environments. In this study, we used cucumber seedlings as experimental material, employing dark pretreatment combined with red, blue, and white light irradiation. This approach systematically investigated changes in stomatal aperture, photosynthetic parameters (net photosynthetic rate, stomatal conductance, and transpiration rate), and instantaneous water use efficiency (iWUE) under drought stress. Furthermore, transcriptome analysis during 0–6 h of BL treatment was performed to uncover the transcriptomic characteristics and key candidate regulatory genes underlying the crosstalk between BL and drought signals in modulating stomatal movement. Our findings aim to elucidate how plants coordinate light and drought signals at the transcriptional level, with a focus on aquaporins, to fine-tune stomatal behavior, finally providing a new potential target for the synergistic improvement of crop photosynthetic efficiency and drought resistance.

## 2. Results

### 2.1. Effects of Light Quality on Photosynthetic Parameters and Stomatal Aperture Under Non-Drought and Drought Conditions

**Figure 1:**
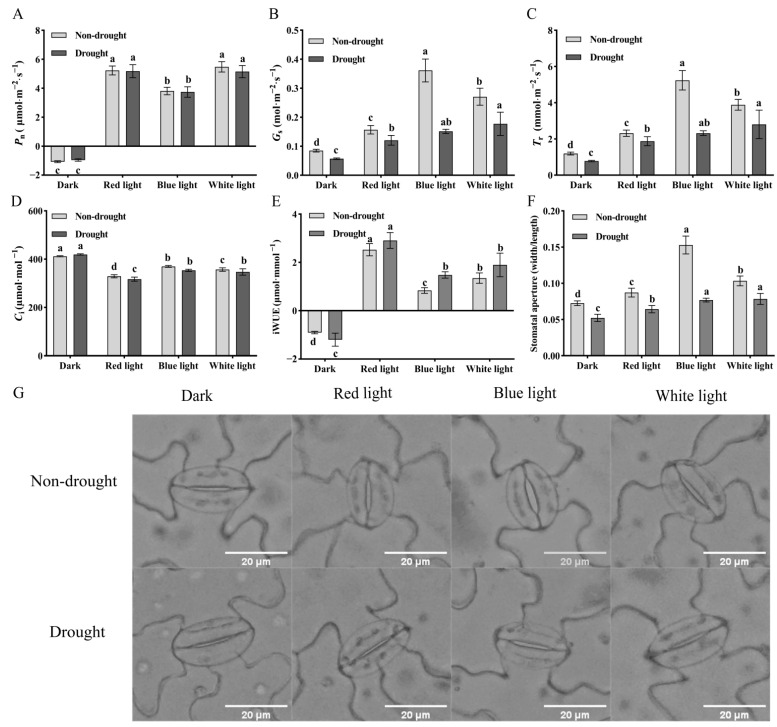
Effects of light quality on photosynthetic parameters and stomatal aperture of cucumber seedlings under drought and non-drought conditions. (**A**–**E**) Effects of light quality on *P*_n_, *G*_s_, *C*_i_, *T*_r_, and iWUE under drought and non-drought conditions. (**F**,**G**) Effects of light quality on stomatal aperture (width/length) under drought and non-drought conditions. Letters above the error bars are the significant differences according to post hoc Duncan’s multiple comparison tests (*p* < 0.05); the significant differences are compared within each drought condition.

According to the two-way ANOVA results (Table S1), light quality, drought conditions, and their interaction significantly influenced several parameters in cucumber seedlings, including instantaneous water use efficiency (iWUE), stomatal aperture, and photosynthetic parameters, such as stomatal conductance (*G*_s_), intercellular CO_2_ concentration (*C*_i_), and transpiration rate (*T*_r_). This finding suggested that the effect of light quality on these indicators depended on the drought conditions. In addition, there was a significant main effect of light quality, but no significant light quality × drought interaction on net photosynthetic rate (*P*_n_). Specifically, under non-drought conditions, *P*_n_ under RL and WL showed no significant differences, and both were significantly higher than those under BL (Figure 1A). In contrast, *G*_s_, *T*_r_, and *C*_i_ were all highest under BL, followed by WL and RL (Figure 1B–D). After drought stress, *P*_n_ did not differ significantly across light treatments (Figure 1A), while *G*_s_ and *T*_r_ were significantly reduced, with the most pronounced reductions observed under BL (*G*_s_ decreased by 58.1% and *T*_r_ by 55.7%) (Figure 1B,C). Meanwhile, *C*_i_ under RL and BL decreased significantly after drought stress but showed no significant change relative to the non-drought condition under WL (Figure 1D). Consequently, iWUE increased significantly under drought stress compared with non-drought conditions, showing increases of 15.3%, 76.7%, and 40.6% for RL, BL, and WL, respectively (Figure 1E). As shown in Figure 1F,G, after a 24 h dark pretreatment, all cucumber seedlings exhibited closed stomata. After 3 h of light exposure, all treatments exhibited increased stomatal apertures. Notably, under non-drought conditions, BL treatment resulted in markedly larger stomatal apertures compared with the other light treatments. Following drought stress, significant stomatal closure was observed, and the stomatal apertures under RL, BL, and WL treatments decreased by 26.3%, 50.0%, and 24.0%, respectively, compared with non-drought conditions. Given that the effects of light quality on *G*_s_ and stomatal aperture depended on water status, and that the pronounced sensitivity of BL-mediated stomatal movement to drought stress was evident, we decided to focus on BL for subsequent molecular investigations.

### 2.2. Transcriptome Sequencing and Analysis of Differentially Expressed Genes (DEGs)

#### 2.2.1. Statistics of Sequencing Data Output and Assembly Results

To discern the molecular mechanism of stomatal movement under BL with or without drought, mRNA transcriptome sequencing of cucumber leaf tissues was performed using the DNBSEQ-T7 high-throughput sequencing platform (BGI MGI). Leaf samples were collected 0, 0.5, 1, 2, 3, and 6 h after BL treatment under both non-drought and drought conditions. The resulting data were of sufficient coverage and quality for subsequent analysis (see Tables S2 and S3 for detailed metrics).

#### 2.2.2. Analysis of DEGs

**Figure 2:**
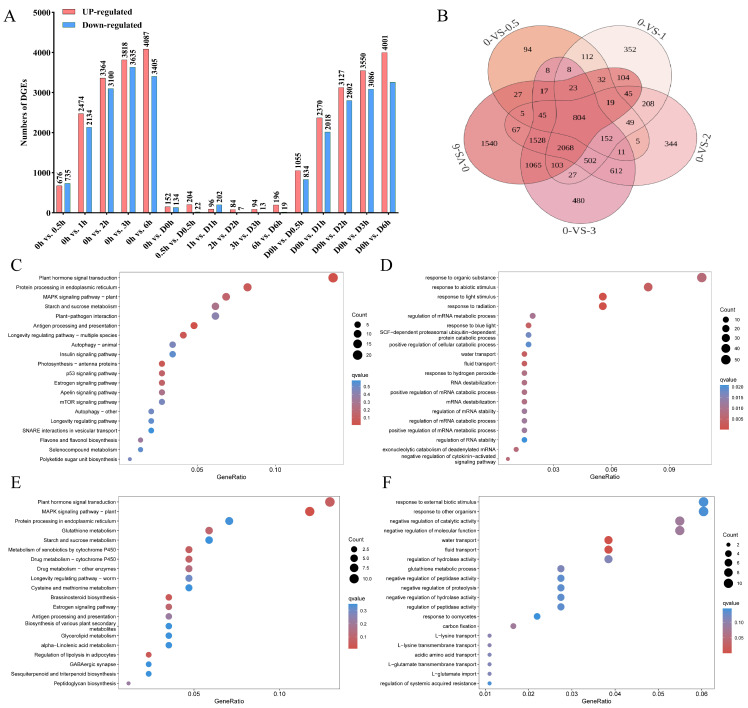
DEGs in each comparison group and enrichment analysis of BL-responsive genes and drought stress-responsive genes. (**A**) DEGs in each comparison group. (**B**) Venn analysis of overlapping DEGs between the five comparative groups (0 h vs. 0.5 h, 0 h vs. 1 h, 0 h vs. 2 h, 0 h vs. 3 h, and 0 h vs. 6 h). (**C**) Bubble plot of KEGG enrichment results for BL-responsive genes. (**D**) Bubble plot of GO enrichment results for BL-responsive genes. (**E**) Bubble plot of KEGG enrichment results for DEGs in 0 h vs. D0 h comparison. (**F**) Bubble plot of GO enrichment results for DEGs in 0 h vs. D0 h comparison.

To screen for candidate genes responsive to BL and/or drought stress that may be involved in regulating stomatal movement, we performed differential expression analysis on the quantified transcriptome data. Based on the threshold of adjusted *p*-value (*p*_adj_ < 0.05) and |log_2_FC| > 1, DEGs under different sample states were screened. Because these DEGs were identified from bulk whole-leaf transcriptome data, they do not clearly reveal the cell-type origin of the expression changes. As shown in Figure 2A, the effect of BL on gene expression was significantly time-dependent. Under non-drought conditions, compared with the dark treatment, the number of BL-induced DEGs increased significantly with the extension of treatment time. A total of 1411, 4608, 6464, 7453, and 7492 DEGs were identified in the comparisons of 0 h vs. 0.5 h, 0 h vs. 1 h, 0 h vs. 2 h, 0 h vs. 3 h, and 0 h vs. 6 h, respectively. Under drought conditions, compared to the dark treatment, BL treatment for 0.5, 1, 2, 3, and 6 h yielded 1889, 4388, 5929, 6636, and 7258 DEGs, respectively. Moreover, the comparison between drought and non-drought treatments at the corresponding time points under BL identified 286, 226, 298, 91, 107, and 215 DEGs at 0, 0.5, 1, 2, 3, and 6 h, respectively.

#### 2.2.3. Analysis of Functional Enrichment of DEGs

**Figure 3:**
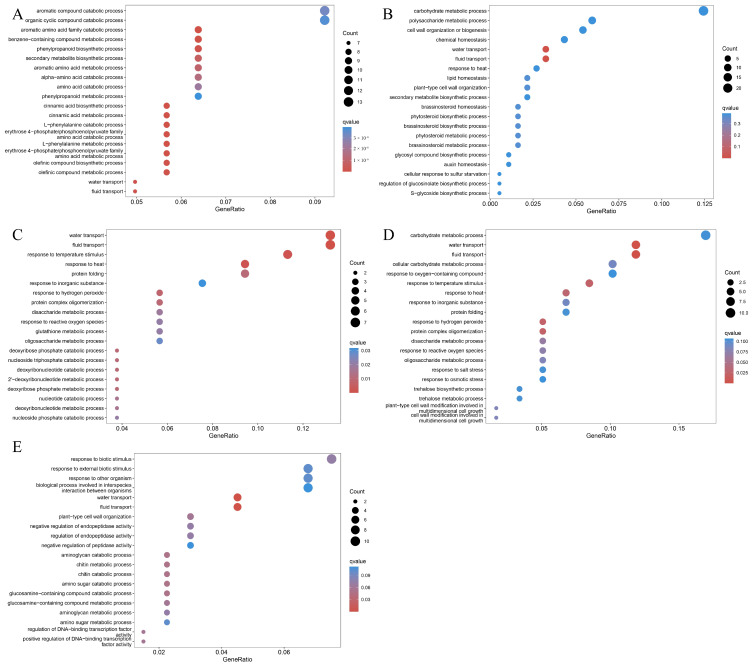
Enrichment analysis of drought stress-responsive genes under BL conditions. (**A**–**E**) Bubble plot of GO enrichment results for DEGs in 0.5 h vs. D0.5 h, 1 h vs. D1 h, 2 h vs. D2 h, 3 h vs. D3 h, and 6 h vs. D6 h, respectively.

To investigate the regulatory mechanism of BL on stomatal movement, we analyzed the DEGs between dark and BL irradiation for different durations (0.5, 1, 2, 3, and 6 h). Venn diagram analysis showed that among the five time-point comparison groups (0 h vs. 0.5 h, 0 h vs. 1 h, 0 h vs. 2 h, 0 h vs. 3 h, and 0 h vs. 6 h), there were 804 DEGs that were consistently responsive to BL, which are critical genes in the BL signaling pathway (Figure 2B). Functional enrichment analyses (KEGG and GO) of these core BL-responsive genes revealed that they were significantly enriched in multiple key pathways including “Plant hormone signal transduction”, “MAPK signaling pathway-plant”, “Photosynthesis-antenna proteins”, “Antigen processing and presentation” (Figure 2C), and biological processes such as “response to light stimulus”, “response to radiation”, “water transport”, and “fluid transport” (Figure 2D). To further investigate the response mechanism to drought stress, we identified drought-induced DEGs under dark conditions and performed enrichment analysis on these genes. The results showed that the DEGs induced by drought stress were also significantly enriched in the “Plant hormone signal transduction” and “MAPK signaling pathway-plant” (Figure 2E), and the biological processes including “water transport” and “fluid transport” (Figure 2F). This suggests that there are overlapping pathways in cucumber’s responses to BL and drought, and these two factors may jointly regulate the key physiological process of “water/fluid transport” by coordinately regulating the “Plant hormone signal transduction” and “MAPK signaling pathway”. Furthermore, to reveal the transcriptional-level interaction between BL and drought, we analyzed the DEGs between BL under drought conditions (BD) and BL at each time point and found that only “water/fluid transport” biological process was consistently and significantly enriched throughout the 6 h time course (Figure 3). This suggests that “water/fluid transport” may be a key regulatory node where BL signaling and drought signaling pathways converge.

#### 2.2.4. Analysis of Core Genes in “Water/Fluid Transport” Biological Processes

**Figure 4:**
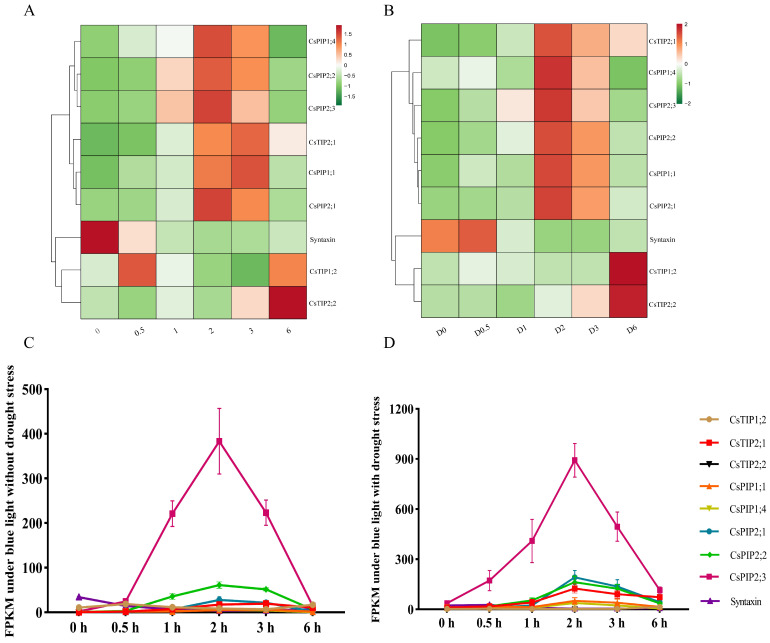
Heatmap and Fragments Per Kilobase of transcript per Million fragments mapped (FPKM) analyses of core water-transport gene expression. (**A**,**B**) Expression patterns under BL and BD treatments at 0, 0.5, 1, 2, 3, and 6 h. (**C**,**D**) FPKM quantification under BL and BD treatments at 0, 0.5, 1, 2, 3, and 6 h.

We further analyzed key genes enriched in the “water/fluid transport” pathway during the 0–6 h period, including aquaporins *CsTIP1;2*, *CsTIP2;1*, *CsTIP2;2*, *CsPIP1;1*, *CsPIP1;4*, *CsPIP2;2*, *CsPIP2;1*, *CsPIP2;3*, and the vesicle trafficking regulator CsaV3_3G038070 (putative Syntaxin). Most genes exhibited a transient upregulation followed by a decline under both BL and BD treatments (Figure 4A,B). Expression pattern analysis revealed that *CsPIP2;3* showed a rapid induction during 0–2 h and a subsequent reduction during 2–6 h of BL exposure, with significantly higher expression levels under BD than under BL (Figure 4C,D). In contrast, other aquaporins such as *CsTIP1;2* and *CsPIP2;1* showed weaker responses to BL and BD treatments. These results identify *CsPIP2;3* as a prime candidate that mediates the integration of BL and drought signals through the regulation of water transport.

### 2.3. Analysis of Genes Related to Cucumber Plasma Membrane Intrinsic Aquaporin CsPIPs

#### 2.3.1. qRT-PCR Validation

To further confirm the accuracy of transcriptomic data, we selected 10 CsPIP family genes for qRT-PCR verification. Overall, the expression levels detected by qRT-PCR were highly consistent with those from the transcriptome (Figure S1), indicating that the RNA-seq data were reliable.

#### 2.3.2. Analysis of BL-Induced Expression of the CsPIPs Family

**Figure 5:**
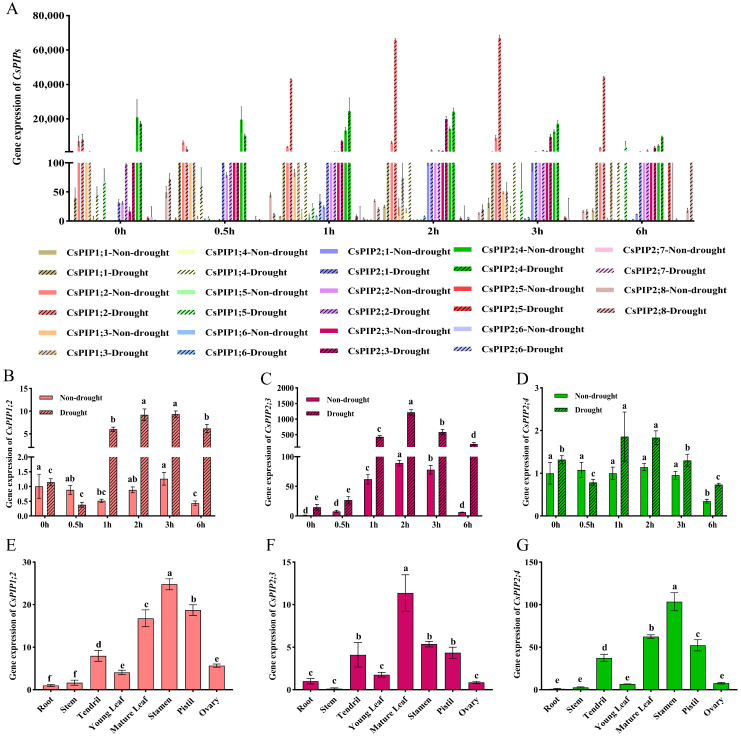
Expression patterns of *CsPIPs* in response to BL under non-drought and drought conditions and in different tissues. (**A**) Expression of all *CsPIPs* family members under BL treatment with non-drought and drought conditions. (**B**–**D**) Relative expression levels of *CsPIP1;2*, *CsPIP2;3*, and *CsPIP2;4*, respectively, under BL treatment with non-drought and drought, and the significant differences are compared within each drought condition (letters above the error bars are the significant differences according to post hoc Duncan’s multiple comparison tests (*p* < 0.05)). (**E**–**G**) Relative expression levels of *CsPIP1;2*, *CsPIP2;3*, and *CsPIP2;4*, respectively, in different cucumber tissues.

**Figure 6:**
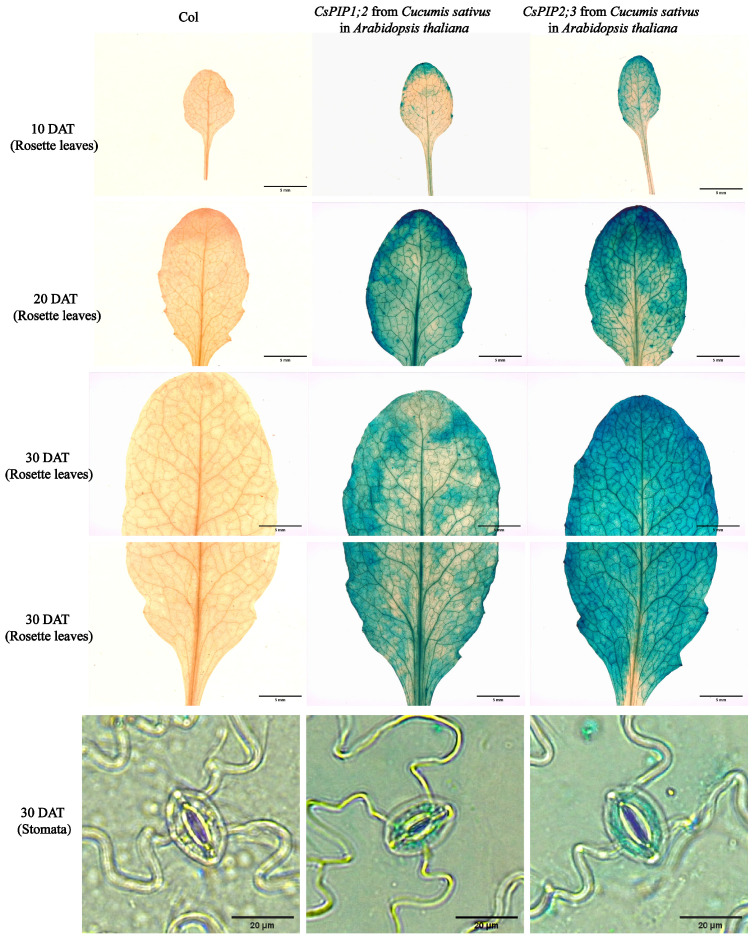
Expression patterns of *CsPIP1;2* and *CsPIP2;3* in rosette leaves and stomata.

To analyze the response of *CsPIPs* to BL under non-drought and drought conditions, the gene expression levels of *CsPIPs* under both conditions were detected by qRT-PCR. As shown in Figure 5A, *CsPIP1;2*, *CsPIP2;3*, and *CsPIP2;4* exhibited higher expression under BL and BD than other *CsPIPs*. Among them, *CsPIP1;2* only responded to BL under drought stress (Figure 5B). The expression of *CsPIP2;3* under BL and BD treatments showed an initial increase and a subsequent decline over time, and its expression under BD was higher than that under BL (Figure 5C). In addition, the expression of *CsPIP2;4* under BL showed a decreasing trend over time, while under BD it exhibited an initial increase followed by a decrease. Overall, relatively modest expression fluctuations were observed under both treatments (Figure 5D). Tissue-specific expression analysis showed that *CsPIP1;2* exhibited the highest expression level in stamens, followed by pistils, mature leaves, and tendrils (Figure 5E). Similarly, *CsPIP2;4* also showed the highest expression level in stamens, followed by lower levels in mature leaves, pistils, and tendrils (Figure 5G). In contrast, *CsPIP2;3* had the highest expression level in mature leaves, with moderate expression observed in pistils and tendrils (Figure 5F). Notably, all three genes displayed consistently low expression levels in roots, stems, young leaves, and ovaries (Figure 5E–G). In addition, we analyzed the spatial expression pattern of *CsPIP1;2* and *CsPIP2;3* in the rosette leaves and stomata using GUS reporter genes driven by their respective promoters (Figure 6). GUS staining showed that Pro*PIP1;2*:GUS and Pro*PIP2;3*:GUS resulted in strong staining in the mesophyll and veins. A close-up view of the stomata showed even staining in the guard cells. These findings showed that *CsPIP1;2* and *CsPIP2;3* may play an essential role in water transport within stomata and vascular bundles.

**Figure 7:**
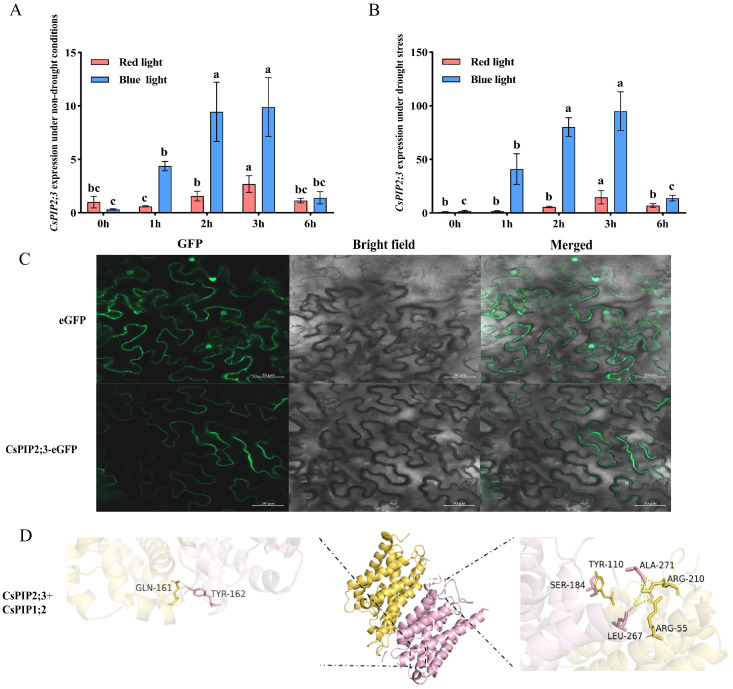
Analysis of BL-specific response, distribution of CsPIP2;3-GFP fusion protein, and prediction of interaction protein binding sites for CsPIP2;3. (**A**,**B**) Relative expression of *CsPIP2;3* in response to RL and BL under non-drought and drought conditions. Letters above the error bars are the significant differences according to post hoc Duncan’s multiple comparison tests (*p* < 0.05); the significant differences are compared within each light quality. (**C**) Subcellular distribution of CsPIP2;3-GFP fusion protein in tobacco epidermal cells. (**D**) The specific amino acid residues involved in the interactions between CsPIP2;3 and CsPIP1;2. Yellow residues represent CsPIP1;2, and pink residues represent CsPIP2;3.

Furthermore, the relative expression of *CsPIP2;3* exhibited a trend of initial upregulation followed by downregulation during RL and BL treatment (0, 0.5, 1, 2, 3, and 6 h) under both non-drought and drought conditions, peaking at 3 h post-light exposure. The relative expression under drought stress was significantly higher than that under non-drought conditions. In addition, the inductive effect of BL on *CsPIP2;3* was markedly stronger than that of RL (Figure 7A,B), highlighting its specific responsiveness to BL, the most potent light quality for regulating stomatal dynamics. Finally, transient expression-based subcellular analysis in *N. benthamiana* leaf epidermal cells indicated that CsPIP2;3 was predominantly distributed at the plasma membrane (Figure 7C). The plasma membrane is the primary site where this protein regulates water flux across the cell membrane in guard cells and other leaf cell types closely associated with stomatal movement.

#### 2.3.3. Analysis of Cis-Acting Elements and Prediction of Interacting Proteins of CsPIP2;3

Analysis using PlantCARE revealed that the promoter sequence of CsPIP2;3 contains various plant hormone-responsive elements, including ABA-responsive elements (ABRE, ABRE3a, and ABRE4), an auxin-responsive element (AuxRE), an ethylene-responsive element (ERE), and an SA-responsive element (TCA-element). It also contains light-responsive elements such as AE-box, Box 4, G-Box, and I-box, as well as stress-responsive elements including anaerobic induction-responsive element (ARE), drought-inducibility element (MBS), multiple stress-responsive element (STRE), transcription factor MYB binding sites (Myb, MYB), and MYB and bHLH binding sites (Myc, MYC). In addition, there is an element involved in circadian rhythm regulation (circadian) (Table S4). These findings suggest that *CsPIP2;3* has the potential to serve multiple functions and may be regulated by various plant hormones in a cell-type-specific manner. Furthermore, it may contribute to the coordinated integration of multiple responses in leaf tissues, including responses to stresses, light signals, and the regulation of circadian rhythms.

To predict whether CsPIP2;3 interacts with CsPIP1;2, we retrieved the protein sequences from UniProt and predicted the protein–protein interactions with HDOCK. HDOCK predictions indicated that CsPIP2;3 and CsPIP1;2 may form stable complexes. The predicted models exhibited the highest confidence score (confidence score = 1.000) and high conformational prediction accuracy (ligand RMSD < 0.6 Å). The docking scores suggest that these theoretically predicted interactions are highly feasible. Furthermore, the predicted binding affinity for CsPIP2;3 and CsPIP1;2 (docking score = −702.65) was slightly strong, as indicated by the negative score (Table 1). To identify the specific amino acid residues in CsPIP1;2 that are putatively involved in the interaction with CsPIP2;3, we utilized PyMOL 3.1.0 (Warren DeLano, South San Francisco, CA, USA) to visualize the protein–protein binding sites. Ser-184, Tyr-162, and Tyr-110, among others, were identified as probable binding sites of CsPIP2;3-CsPIP1;2 complex (Figure 7D). Notably, all the above interaction patterns are only theoretical predictions based on bioinformatic analysis; in situ protein localization assays and in vivo protein–protein interaction assays (e.g., co-immunoprecipitation or BiFC) are required to verify their actual physiological relevance in plants.

## 3. Discussion

### 3.1. Under Non-Drought Conditions, RL Enhanced Photosynthetic Rate, While BL Promoted Stomatal Opening

Currently, the effects of RL and BL on plants can be temporally categorized into two types: the instantaneous effects and the long-term adaptive responses on the photosynthetic apparatus [24]. This study mainly explored the instantaneous effects of light quality on the photosynthetic induction of cucumber seedlings. Previous studies have found that the photosynthetic efficiency of pure BL is lower than that of pure RL [25]. As a result, when leaves are irradiated with the same light intensity simultaneously, the photosynthetic rate (*P*_n_) under RL is typically higher than that under BL [24]. This study found that *P*_n_ under BL was significantly lower than that under RL and white light (WL) (Figure 1A), which is consistent with previous research results on the instantaneous effects of RL and BL on photosynthetic induction. In contrast, the stomatal aperture and the indices related to stomatal aperture, i.e., *G*_s_ and *T*_r_, were all significantly higher under BL than under RL (Figure 1B,C,F). This result is consistent with the findings of studies on cucumber by Wang et al. [26] and Liang et al. [27]. However, the above studies mainly focused on the impact of long-term light quality treatment on *G*_s_. This study further found that even under short-term treatment, the *G*_s_ and stomatal aperture under BL treatment were significantly higher than those under RL and WL treatments (Figure 1B,G). This indicates that both short-term and long-term BL treatment can stably and effectively increase *G*_s_, and this effect is stronger than that of RL and WL. This result confirms the classic mechanism by which BL rapidly and efficiently induces stomatal opening through phototropins (Phot) [28]. Studies have shown that BL is more efficient than RL in inducing stomatal opening: the stomatal response induced by BL, which can reach saturation under a low photon flux density of 5–10 µmol·m^−2^·s^−1^, is specific to guard cells and independent of mesophyll photosynthesis [29]. In contrast, RL-induced stomatal opening mainly relies on the indirect regulation of mesophyll cell photosynthesis or phytochromes (such as phyB). This process involves a long signal transmission path and complex steps, and requires higher light intensity for activation, thus having lower efficiency [11]. At the same time, RL triggers a negative regulatory mechanism for guard cell stomatal opening to prevent excessive stomatal opening and water loss [30,31,32]. According to Li et al., the transcriptional level of *MPK11* in guard cells is upregulated by both WL and RL, which inhibits stomatal opening. However, the larger stomatal aperture in *mpk11* mutants was not observed under BL, indicating that *MPK11* only plays a negative regulatory role in RL- and WL-induced stomatal opening rather than in BL-induced stomatal opening [30]. This is also the reason why the stomatal aperture under WL and RL treatments is smaller than that under BL treatment in this study. Consequently, this fundamental difference in signaling efficiency and regulation under well-watered conditions laid the foundation for the strikingly divergent stomatal responses we observed under drought stress.

While the stomatal response to BL was pronounced, the discrepancy between the highest *G*_s_ and the lowest *P*_n_ under BL among the light treatments (Figure 1) indicates that the primary constraint on carbon assimilation resides within the mesophyll rather than at the stomatal level. Photosynthesis is intrinsically a mesophyll-driven process [33], and mesophyll conductance (*g*_m_) is widely considered an important limiting factor for photosynthesis [34]. The reduced *P*_n_ under BL, despite maximized stomatal opening, can be attributed to mesophyll-specific constraints. For instance, BL exposure is known to induce chloroplast avoidance movement in the mesophyll, potentially reducing the surface area of chloroplasts appressed to the intercellular airspace and decreasing photosynthetic light absorption. This reduced surface area of chloroplasts exposed to intercellular air spaces may affect the CO_2_ diffusion path to Rubisco carboxylation sites and inhibit photosynthetic rate [35]. Furthermore, BL may directly impair CO_2_ diffusion efficiency in the mesophyll by downregulating carbonic anhydrase (CA) activity or affecting aquaporin functionality, thereby inhibiting *P*_n_ [36].

### 3.2. Stomatal Limitation Is the Core Response Strategy in the Early Stage of Drought

Stomatal limitation is a core response strategy in the early stage of drought, which alleviates water stress by rapidly reducing transpirational water loss [37,38]. In this study, the *G*_s_, Tr, and stomatal aperture under all light quality treatments were significantly reduced after drought stress, which is consistent with previous research results [39,40]. Our study indicated that the effect of light quality on these indicators depended on the water conditions (Table S1). Specifically, *G*_s_ under BL decreased by 58.1% compared with the non-drought condition, which was much higher than the decrease under RL and WL after drought stress (Figure 1B). This suggests that stomata under BL treatment are more sensitive to drought stress. However, after drought stress, *G*_s_ under BL remained comparable to that under WL and was significantly higher than those under RL (Figure 1B), suggesting that the promoting effect of BL on stomatal opening is partially retained even under drought conditions. This complex behavior of stomata under BL highlights its unique and pivotal role in mediating plant responses to drought, a role that might be intrinsically linked to the hormone ABA. Researchers observed elevated ABA levels in the leaves of well-watered *Arabidopsis cry* mutants. This finding suggests that the absence of CRY-mediated BL perception results in increased ABA accumulation and reduced transpiration [41]. Other studies have also indicated that light signaling pathways intersect with ABA regulatory pathways at multiple junctions [42,43]. Indeed, research on *A*. *thaliana* has demonstrated that under drought stress, BL-induced stomatal opening is strongly inhibited by ABA, ultimately triggering stomatal closure [44]. Conversely, under well-watered conditions, BL suppresses ABA signaling to promote stomatal opening [45]. The critical question then becomes, at which molecular node(s) are these antagonistic signals integrated to execute the precise control of stomatal aperture? Our data point to the process of water transport as a central hub.

### 3.3. The “Water Transport” Biological Process May Be a Common Hub Through Which BL and Drought Signals Regulate Stomatal Movement

Stomata could respond quickly and sensitively to hydraulic signals in response to the external environment, ultimately achieving near-homeostasis in leaf water potential [46,47]. This response is achieved through rapid and directional water transport between the guard cells and the surrounding cells (such as epidermal cells) [48]. In this study, we found that under non-drought conditions, core BL-responsive genes were significantly enriched in the biological process “water transport” (Figure 2D). In addition, drought-responsive genes were also significantly enriched in this biological process under dark (Figure 2F) and under BL (Figure 3). These results indicate that the “water transport” process may be the core hub where BL and drought signaling pathways intersect and jointly regulate stomatal movement. Next, we analyzed the key genes enriched in the “water transport” process, with a focus on PIPs in cucumber, and identified *CsPIP2;3* as a key candidate gene. This gene is not only rapidly and strongly induced by BL, with its expression further enhanced under drought stress (Figure 4C,D), but is also predominantly expressed in mature leaves, where water exchange is most active (Figure 5F and Figure 6). In addition, a transient expression assay verified the predominant plasma membrane localization of CsPIP2;3, which is consistent with the conserved subcellular distribution of PIP family aquaporins. This localization pattern suggests its potential direct role in regulating transmembrane water transport across cucumber leaf cell membranes (Figure 7C). Notably, this *N. benthamiana* transient expression assay only serves as preliminary verification with inherent limitations of heterologous expression, and the precise localization of CsPIP2;3 in cucumber will be further validated via more rigorous methods. Furthermore, its response specificity to BL significantly surpasses that to RL, consistent with its putative core function in BL-mediated stomatal movement (Figure 7A). PIPs play a role in regulating the selective transport of water molecules and small molecular substances across plant cell membranes to maintain water dynamic balance and osmotic balance, making them putative participants in the regulation of guard cell turgor pressure [49]. Ding et al. found that *ZmPIP2;5* can enhance the sensitivity of stomatal closure to water deficit. Under sufficient water conditions, overexpression of *ZmPIP2;5* leads to an increase in stomatal conductance and aperture. However, under water stress, overexpression of *ZmPIP2;5* causes a reduction in stomatal conductance and leaf gas exchange [50]. Wang et al. demonstrated that *AtPIP2;1* facilitates the entry of CO_2_ into guard cells, accelerating the production of intracellular HCO_3_^−^, which in turn enhances the activity of the SlAC1 anion channel, a key player in stomatal closure [51]. In conclusion, we speculate that *CsPIP2;3* may be a key gene that integrates BL and drought signals to regulate water transport, thereby directly mediating stomatal movement at the guard cell level. However, this complex mechanism requires further investigation.

Leaf hydraulic conductance (K_leaf_), the core metric describing leaf water transport efficiency, can be simplified into two distinct components: xylem hydraulic conductance (K_x_) and out-of-xylem hydraulic conductance (K_ox_) [52]. Unlike Kx, which is determined by fixed lignified xylem structures and cannot be dynamically regulated in response to environmental signals, Kox is modulated by living cells along the radial transport path, including vascular parenchyma, BSCs, and MCs, making it the primary regulatory node of K_leaf_ in response to internal and environmental cues [52,53]. The radial water transport path corresponding to Kox follows a conserved core route in plant leaves: from the minor vein xylem, through BSCs and mesophyll tissues, and finally to the stomata [53]. The efficiency of this path directly determines K_leaf_, and high-efficiency K_leaf_ is widely recognized as the fundamental basis for maintaining stomatal opening [54]. The BSCs, which form a continuous layer tightly enveloping the xylem throughout the leaf, act as a selective barrier to water and solutes entering the mesophyll. This cell layer is widely recognized as a major signal-perceiving “valve” in series with stomata, which modulates leaf radial K_leaf_ and thereby regulates radial water flow across the transpiring leaf [55]. BL has been shown to increase the K_leaf_ of the entire leaf through the regulation of PIPs [56]. This effect might be mediated by BSCs, which possess a BL signal transduction pathway similar to the one that induces stomatal opening. In this model, the phot1 and phot2 in BSCs perceive the BL, which, through a kinase-dependent pathway, in turn activates plasma membrane H^+^-ATPase (AHA2), resulting in hyperpolarization of the BSCs and the acidification of the xylem sap. This acidification increases the water permeability of the BSC plasma membrane. This effect is presumed to stem from enhanced permeability of aquaporins, which underpins the elevated osmotic water permeability coefficient (P_f_) of individual BSCs and, in turn, contributes to the increased K_leaf_ [55,57]. Under drought stress, ABA translocated within the xylem sap can be perceived at the plasma membrane of BSCs. This perception event triggers the functional inactivation of AQPs and thereby reduces the water permeability of BSC membranes for transcellular water transport into the mesophyll tissue [21,53]. BSCs, as the core gatekeepers of leaf radial water transport, drive a marked decrease in Kox through their reduced water permeability, leading to a significant decline in K_leaf_. The consequent decline in K_leaf_ accelerates dehydration of the mesophyll, which further stimulates de novo ABA biosynthesis and may ultimately drive stomatal closure [52]. Mesophyll tissue is the core downstream compartment of BSC-controlled water transport. After exiting the bundle sheath, leaf water must pass through multiple layers of MCs before finally reaching the substomatal cavity, where evaporation occurs. This entire transport process occurs exclusively within the mesophyll tissue, making MCs the core functional compartment for the full leaf water transport pathway [58,59]. Under most conditions, the apoplastic pathway (water movement through cell walls and intercellular spaces, an aquaporin-independent transport route) in the mesophyll provides the majority of conductance outside the bundle sheath [58]. Studies have shown that stomatal movement is closely related to plant hydraulic characteristics. Both stomatal opening and the increase in K_leaf_ occur in the early morning when the proportion of BL is relatively high, making stomatal opening match the increase in leaf water flux. Therefore, BL may indirectly regulate stomatal movement by modulating plant hydraulic characteristics via PIPs, and the PIPs specifically localized in the BSCs may play a dominant role in this regulatory process. These results suggest that, in addition to the known effect of BL on the hydroactive opening response of stomata, it can also affect stomatal movement by enhancing the xylem–epidermis water supply [60]. Additionally, *CsPIP2;3* is positioned as a key integrator of BL and drought signals. It may orchestrate stomatal movement through a dual mechanism: a direct pathway by regulating water flux across guard cell membranes, and an indirect pathway by modulating the leaf’s hydraulic capacity to supply water.

**Figure 8:**
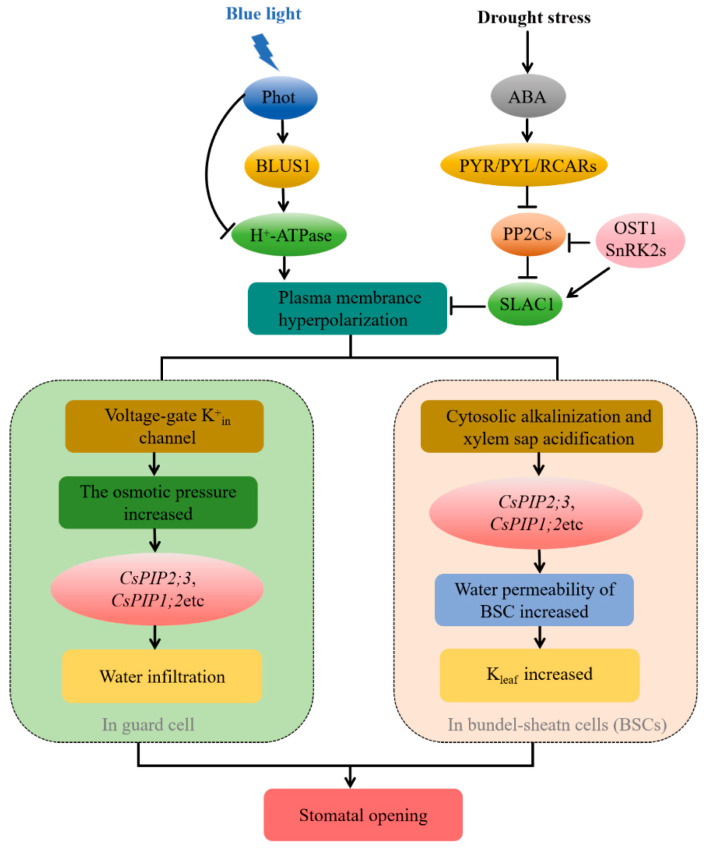
Mechanistic model of stomatal movement regulation by BL and drought. This model illustrates the multicellular coordinated regulatory network of stomatal movement mediated by BL signaling and drought-induced ABA core signaling transduction pathway.

Furthermore, whether in dark or BL conditions, the expression of *CsPIP2;3* under drought conditions was significantly higher under drought than under non-drought conditions (Figure 4C,D). This drought-induced upregulation of *CsPIP2;3* likely occurs within the broader context of ABA signaling. Studies have shown that drought-induced ABA not only regulates stomatal aperture directly but also modulates whole-plant hydraulic characteristics, aquaporin activity, and stomatal density [61]. Crucially, previous studies have established that PIPs play an important role in ABA-induced stomatal closure. Grondin et al. found that the *AtPIP2;1* knockout mutant has a defect in stomatal closure, and ABA-triggered stomatal closure may require OST1-dependent phosphorylation of PIP2;1 to increase the permeability of guard cells to water and H_2_O_2_ [62]. Rodrigues et al. pointed out that phosphorylated *AtPIP2;1* significantly enhances the permeability of guard cell protoplasts, promotes the diffusion of H_2_O_2_ into guard cells, and thereby mediates ABA- and flg22-induced stomatal closure [63]. Therefore, *CsPIP2;3* may serve as a dual integrator of BL and drought signals, regulating water transport pathways to ultimately achieve modulation of stomatal aperture. Based on our results, we propose a possible regulatory model (Figure 8). This mechanistic model represents a novel contribution to the current understanding of stomatal movement regulation. Unlike previous models that focus on a single signal (either BL or drought), this study establishes a coordinated regulatory network that integrates BL and drought signals through water transport, *CsPIP2;3*, and its potential interaction partners. This model not only fills the knowledge gap of light-drought crosstalk in stomatal control of protected vegetables but also provides a theoretical reference for precise light regulation and drought-resistant breeding in agricultural production, making the research results valuable for practical horticultural applications.

It should be noted that we only performed cell-specific expression verification for the core gene *CsPIP2;3* in this study, and the precise cell-type-specific expression patterns and sub-functionalization of other aquaporin family members remain to be further validated. Meanwhile, the DEGs screening in this study was based on bulk whole-leaf transcriptome sequencing, which has inherent limitations: it averages gene expression signals across thousands of cells in the leaf, thereby masking the cellular heterogeneity and spatial distribution gradients that are critical for plant development and stress adaptation [64]. Owing to this technical constraint, the differentially expressed genes identified in this study only reflect the average expression level across all cell types in cucumber leaves and cannot distinguish the cell-type-specific transcriptional changes in guard cells, MCs, BSCs, and other key cell types that are central to the regulation of stomatal movement and leaf water transport. Even though single-cell RNA sequencing (scRNA-seq) has strengths in resolving cellular heterogeneity, it inherently separates discrete cell populations and creates substantial difficulties for re-integrating data into a unified whole-system understanding. In contrast, interactomics represents a gold-standard approach in plant biology. This approach enables systematic investigation of dynamic multilayered interactions, including mechanosensory signaling and chromatin status remodeling, and may include scRNA-seq data as part of the analysis [65]. Future studies will require new methodologies that integrate spatial, molecular, and cellular biology in situ with scRNA-seq and interactomics to investigate stomatal movement regulated by BL and drought stress, and to fully clarify the complex regulatory networks in the leaf multicellular system [66].

## 4. Materials and Methods

### 4.1. Plant Material and Light Treatment

Cucumber seeds (‘Xintai Mici’) were hydrated for 8 h in deionized water and then germinated in a constant temperature incubator at 28 °C for 1 day in the dark. The germinated seeds were sown in plug trays filled with a mixed substrate of peat–vermiculite–perlite = 3:1:1 (*v*/*v*/*v*) and then transferred to a controlled environment chamber for cultivation. During the plug seedling stage, seedlings were watered with 1/2 strength Hoagland nutrient solution every two days to maintain a stable nutrient supply. The environmental conditions in the chamber were set as follows: the photosynthetic photon flux density (PPFD) at the seedling canopy level was maintained at 200 µmol·m^−2^·s^−1^, the photoperiod was 14 h/10 h, the average day/night temperature was 23/20 °C, and the relative humidity was 50–70%.

**Figure 9:**
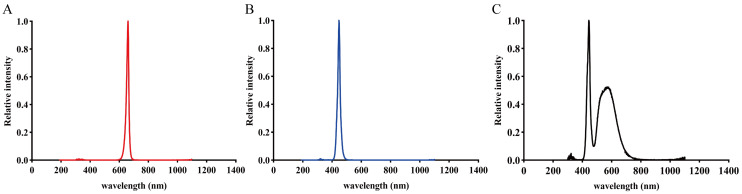
Spectral distribution of red (**A**), blue (**B**), and white (**C**) treatments.

Ten days after the cucumber seedlings emerged, they were transplanted into plastic pots filled with Hoagland nutrient solution for hydroponic culture (Figure S2A), and the nutrient solution was renewed every three days. Nine days after transplantation, seedlings were first subjected to 24 h dark adaptation and then divided into six groups for the following treatments: non-drought and drought treatments were conducted under RL (λmax = 660 nm), BL (λmax = 450 nm), and white light (Figure 9), with drought treatment already initiated during the 24 h dark adaptation period (while non-drought treatments received no drought application during this phase). Drought treatment involved 5% (*w*/*v*) polyethylene glycol (PEG-6000) to simulate drought stress (Figure S2B–D). According to the equation developed by Michel and Kaufmann [67], the osmotic stress level of 5% PEG-6000 in the solution was −0.05 MPa, which corresponds to mild drought. The light source was a DYNA LED system (Heliospectra AB, Gothenburg, Sweden), with light spectra for each treatment programmed using the manufacturer’s proprietary software. Spectral characteristics were measured using a portable back-illuminated fiber-optic spectrometer model AvaSpec-ULS 2048 XL-EVO (Avantes, Apeldoorn, The Netherlands). The PPFD at the seedling canopy level was maintained at 200 µmol·m^−2^·s^−1^.

### 4.2. Measurement of Leaf Gas Exchange Parameters

Photosynthetic parameters, including the net photosynthetic rate (*P*_n_), stomatal conductance (*G*_s_), intercellular CO_2_ concentration (*C*_i_), and transpiration rate (*T*_r_), were measured by a portable LI-6800 photosynthesis system (LI-COR, Lincoln, NE, USA) equipped with an integrated transparent leaf chamber, after 3 h of treatment under different light qualities with non-drought and drought. iWUE was calculated as the ratio of *P*_n_ to *T*_r_ using the formula: iWUE = *P*_n_/*T*_r_. The second fully expanded true leaf was measured using an LI-6800 system. Chamber conditions were maintained at 500 μmol·s^−1^ airflow, 400 μmol·mol^−1^ CO_2_, 10,000 rpm fan speed, and 65% relative humidity. Five biological replicates were conducted per treatment.

### 4.3. Measurement of Stomatal Parameters

Stomatal morphology was assessed using a modified nail polish impression method. Fully expanded leaves from different light-quality treatments were selected at comparable positions, transparent nail polish was applied to the suitable abaxial surface (avoiding main veins) and allowed to air-dry for 5 min. Leaves were then excised, adhered to transparent tape (polished-side down), and peeled to transfer the imprint onto glass slides. Temporary mounts were examined under an optical microscope at 400× magnification. Five non-overlapping fields per leaf were imaged using a digital capture system. The stomatal aperture (defined as the maximum distance between the inner walls of paired guard cells) and aspect ratio were quantified in ImageJ 1.53e (National Institutes of Health, Bethesda, MD, USA), with 10–11 stomata measured per field (totaling approximately 50–55 apertures per leaf). Five biological replicates were included for each treatment to ensure data reliability.

### 4.4. Transcriptome Sequencing and Analysis

Nine days after transplantation, the seedlings were first subjected to 24 h dark adaptation and then divided into two groups for the following treatments: Non-drought and drought treatments were applied under BL (λmax = 450 nm), with the latter including 5% (*w*/*v*) PEG-6000 to simulate drought stress. The PPFD at the seedling canopy level was maintained at 200 µmol·m^−2^·s^−1^. Leaf samples were collected at 0, 0.5, 1, 2, 3, and 6 h after BL exposure. Total RNA was extracted from cucumber leaves using the TransZol Up Simple RNA Kit–Simple RNA Extraction Kit (TransGen Biotech, Beijing, China), and the quality and quantity of RNA samples were assessed using a NanoPhotometer spectrophotometer (Implen, Munich, Germany) and the Agilent 2100 bioanalyzer (Agilent, Santa Clara, CA, USA).

Sequencing libraries were generated using NEBNext^®^ Ultra™ RNA Library Prep Kit for Illumina^®^ (NEB, Ipswich, MA, USA) following the manufacturer’s recommendations. Transcriptome sequencing and analysis were performed by MGI using the DNBSEQ-T7 high-throughput sequencing platform. Raw data (raw reads) of fastq format were first processed through Perl scripts. Then, clean data (clean reads) were obtained by removing reads containing adapters, reads containing poly-N, and low-quality reads. At the same time, Q20, Q30, and GC content of the clean data were calculated. All the downstream analyses were based on the clean data. Genome alignment and gene expression quantification were performed based on the Nature Protocol (doi: 10.1038/nprot.2016.095). Firstly, HISAT2 (v2.1.0) software was used to align the reads to the reference genome (version 3 of the cucumber ‘Chinese Long’ genome). Subsequently, StringTie (v2.2.1) and Ballgown (v2.32.0) software were used for gene expression quantification to get the FPKM of each sample. After gene expression quantification, statistical analysis was performed on the expression data to screen for genes with significantly different expression levels under different experimental conditions. The significant DEGs were screened with |log_2_FC| > 1 and *p*_adj_ < 0.05. The screened DEGs were further subjected to systematic functional annotation analysis, including Gene Ontology (GO) functional enrichment analysis and Kyoto Encyclopedia of Genes and Genomes (KEGG) pathway enrichment analysis, and GO terms with an adjusted *p*-value were considered significantly enriched. The expression patterns of the DEGs between different samples were displayed using heat maps.

### 4.5. Expression of Key Genes and Validation of Transcriptome Data

Total RNA was extracted from cucumber leaves using the TransZol Up Simple RNA Kit (TransGen Biotech) following the manufacturer’s instructions, and the first-strand cDNA was synthesized by reverse transcription using the FastKing gDNA Dispelling RT SuperMix (Tiangen, Beijing, China). Real-time quantitative PCR (qRT-PCR) experiments were performed on the ABI 7500 Real-Time PCR System (Applied Biosystems, Foster City, CA, USA) using the TransStart^®^ Green qPCR SuperMix (TransGen Biotech) to detect the expression of *CsPIPs* genes under BL treatment with drought and non-drought stresses. The PCR cycling conditions were as follows: initial denaturation at 95 °C for 30 s; 40 cycles of 95 °C for 5 s and 60 °C for 34 s; followed by a melt curve stage consisting of 95 °C for 15 s, then 95 °C for 1 min, and finally 95 °C for 15 s. Meanwhile, total RNA was extracted from various cucumber tissues (roots, stems, leaves, tendrils, male flowers, female flowers, and ovaries), and cDNA was synthesized to detect gene expression in these tissues. The geometric mean of the expression levels of the housekeeping gene, namely *ubiquitin* (*UBI*), was selected for result calibration. The quantitative results were analyzed by the 2^−△△Ct^ method. Three biological replicates were included, with three technical replicates per biological replicate. All qRT-PCR primers are listed in Table S5.

### 4.6. Transient Expression and Subcellular Distribution Analysis of CsPIP2;3-GFP

The CDS without a stop codon of *CsPIP2;3* was amplified and ligated into a transient expression pSuper1300-EGFP vector with a GFP fluorescent label and a MAS promoter to construct fusion structures with green fluorescent protein (CsPIP2;3-GFP) (Table S5). The recombinant vectors were transformed into *Agrobacterium tumefaciens* GV3101 by the freeze–thaw method. Next, empty pSuper1300-EGFP (Negative control) or pSuper1300-CsPIP2;3-EGFP GV3101 was instantly infiltrated into 4- or 5-week-old tobacco leaves with expression buffer (10 mM MES pH 5.6, 10 mM MgCl_2_, 200 µM acetosyringone). After the infiltrated tobacco plant was cultured in the dark for 24 h and then in low light for 12 h, the GFP signals were detected by a laser scanning confocal microscope (Carl Zeiss, Oberkochen, Germany).

### 4.7. GUS Staining

To generate the ProCsPIP1;2:GUS and ProCsPIP2;3:GUS constructs, the ~2000 bp genomic fragment upstream of the *CsPIP1;2* and *CsPIP2;3* start codon was amplified by PCR using genomic DNA as the template. Then, the fragment was inserted into the HindIII-BamHI sites of the pBI121 vector to drive the β-glucuronidase (GUS) reporter gene. All primers are listed in Table S5. GUS staining analysis was performed on tissues of ProCsPIP1;2:GUS and ProCsPIP2;3:GUS transgenic *A*. *thaliana* using a ready-to-use GUS staining solution (Regen, Beijing, China) as follows: Rosette leaves were collected from plants at 10, 20, and 30 days after transplantation (DAT), while stomata were harvested from 30 DAT plants. The collected tissues were immersed in the GUS staining solution and incubated overnight at 37 °C in a constant-temperature incubator. After staining, the materials were transferred to 70% ethanol and decolorized 2–3 times until the negative control turned white. Finally, the decolorized samples were soaked in fresh 70% ethanol and observed under an optical microscope.

### 4.8. Statistical Analysis

Statistical analyses were performed using JASP (v 0.18.1, JASP Team, University of Amsterdam, Amsterdam, The Netherlands), and data presentation used GraphPad Prism 9 (GraphPad Software, Inc., La Jolla, CA, USA) and Adobe Illustrator 2021 (Adobe Inc., San Jose, CA, USA). Significant differences among values of all the parameters were determined at *p* ≤ 0.05, according to Duncan’s test.

## 5. Conclusions

Light and drought stress, as key environmental factors regulating stomatal movement, often modulate this process in an antagonistic manner. However, the mechanisms through which plants integrate such conflicting signals remain unclear. The results of this study showed that the effect of light quality on parameters related to stomatal opening, such as *G*_s_ and stomatal aperture, depends on the drought conditions. BL promotes stomatal opening, and the BL-mediated stomatal movement exhibits pronounced sensitivity to drought. Transcriptomic GO and KEGG enrichment analyses showed that genes induced by BL and drought are both enriched in “Plant hormone signal transduction” and “MAPK signaling pathway-plant”, and the biological process of “water transport”–indicating that there are overlapping pathways in cucumber’s responses to BL and drought. These two factors may jointly regulate the key physiological process of “water/fluid transport” by coordinately regulating the “Plant hormone signal transduction” and “MAPK signaling pathway”. Subsequently, the analysis of the key genes in the “water transport” process indicated that *CsPIP2;3* responds strongly to BL compared with other genes, with further enhancement under drought stress. Moreover, this gene is predominantly expressed in mature leaves, where water exchange is most active; its plasma membrane localization also suggests a direct role in regulating transmembrane water transport. Additionally, its specific response to BL is notably higher than its response to RL, which aligns with its proposed core function in BL-mediated stomatal movement. Thus, we hypothesize that *CsPIP2;3* serves as a key integrator of BL and drought signals. It likely regulates stomatal movement through the following mechanism: first, a direct pathway that controls water flux across guard cell membranes; second, an indirect pathway that modulates the leaf hydraulic capacity for water supply.

## Figures and Tables

**Table 1:** HDOCK docking results of model 1.

Protein-Protein Interaction	Docking Score	Confidence Score	Ligand RMSD (Å)
CsPIP2;3 + CsPIP1;2	−702.65	1.000	0.56

## Data Availability

The original contributions presented in this study are included in the article/Supplementary Materials. Further inquiries can be directed to the corresponding authors.

## Supplementary Materials

The following supporting information can be downloaded at: https://www.mdpi.com/article/10.3390/ijms27093717/s1.
