# Supplementary material for: Transcriptome Dynamics Provide Insight into the Mechanisms Underlying Cucumber Stomatal Movement Regulated by Blue Light (BL) and Drought Stress
Source: Int J Mol Sci. 2026 Apr 22;27(9):3717. doi: 10.3390/ijms27093717 (PMC13163241; doi:10.3390/ijms27093717)
Supplement: Supplementary file 1 [file ijms-27-03717-s001.zip › ijms-4240677-supplementary.pdf]

Supplementary Material

1 Supplementary Figures

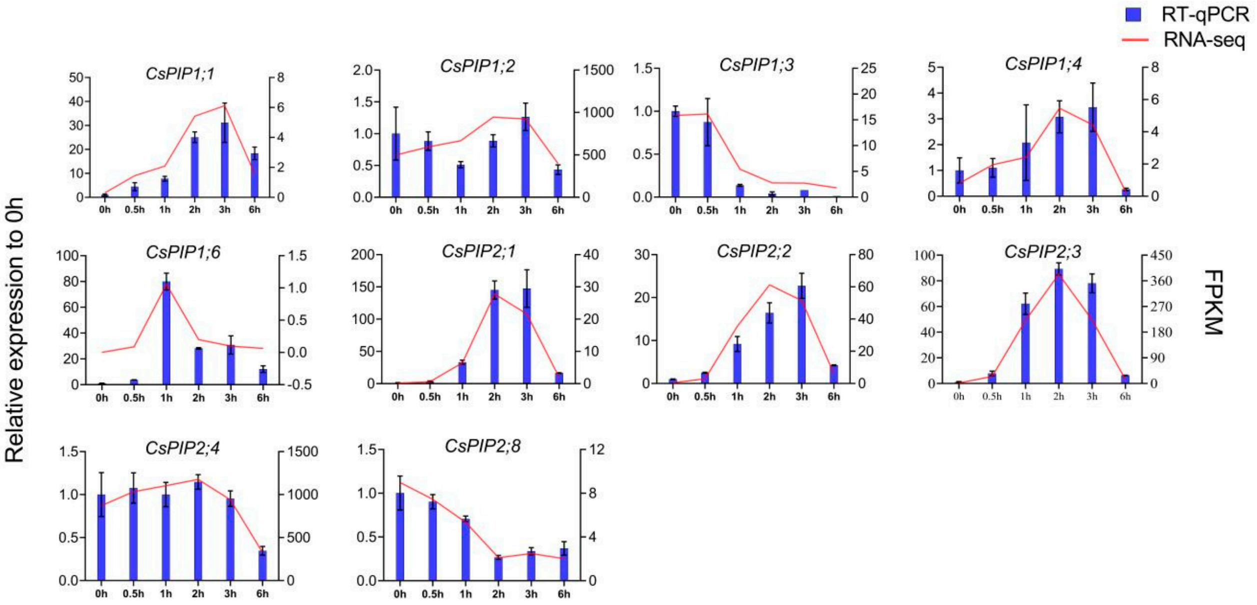

Figure S1. Comparative analysis of RNA-seq results and RT-qPCR results.

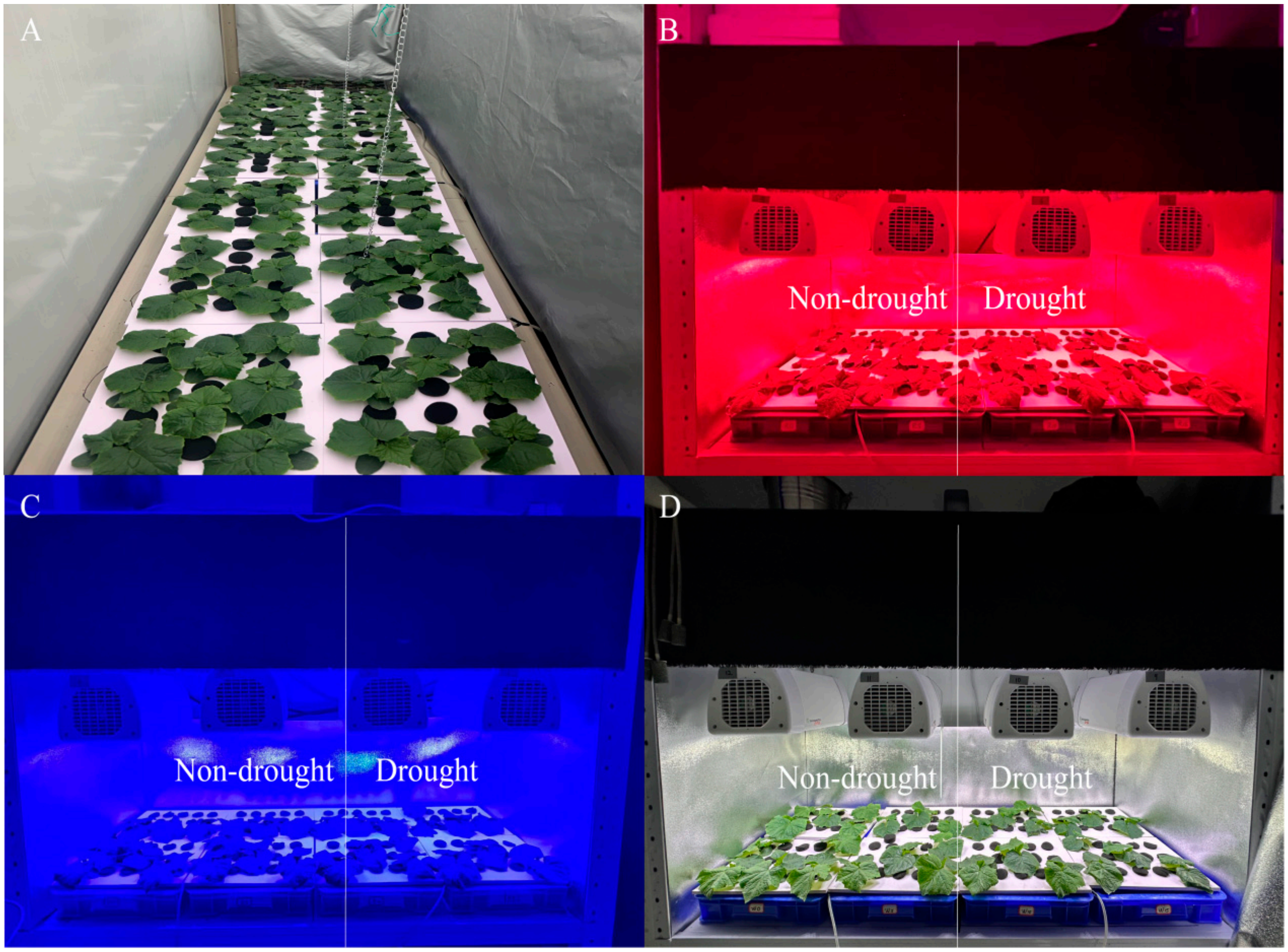

Figure S2. Culture environment and treatment environment of cucumber seedlings. (A) Culture environment; (B-D) Treatment environment.

2 Supplementary Tables

Table S1. Effects of light quality and drought condition on photosynthetic parameters and stomatal aperture in cucumber seedlings based on two-way ANOVA analysis.

Dependent variable	Source	Sum of squares	Degree of freedom	Mean square	F value	P value
Net photosynthetic rate	Corrected model	265.742	7	37.963	309.055	<0.001
	light quality	265.436	3	88.479	720.297	<0.001
	drought condition	0.033	1	0.033	0.269	0.607
	light quality*light quality	0.274	3	0.091	0.743	0.534
Stomatal conductance	Corrected model	0.349	7	0.050	066.122	<0.001
	light quality	0.212	3	0.071	93.828	<0.001
	drought condition	0.084	1	0.084	111.075	<0.001
	light quality*light quality	0.053	3	0.018	23.433	<0.001

Transpiration rate	Corrected model	72.871	7	10.410	59.872	<0.001
	light quality	48.340	3	16.113	92.673	<0.001
	drought condition	14.460	1	14.460	83.164	<0.001
	light quality*light quality	10.071	3	3.357	119.308	<0.001
Intercellular CO_2 concentration	Corrected model	46147.200	7	6592.457	1.9.332	<0.001
	light quality	44665.226	3	14888.409	246.914	<0.001
	drought condition	619.133	1	619.133	10.268	0.003
	light quality*light quality	862.841	3	287.614	4.770	0.007
Instantaneous water use efficiency	Corrected model	77.921	7	11.132	128.177	<0.001
	light quality	75.417	3	25.139	289.469	<0.001
	drought condition	1.089	1	1.089	12.540	0.001
	light quality*light quality	1.415	3	0.472	5.430	0.004
Stomatal aperture	Corrected model	0.034	7	0.005	75.084	<0.001
	light quality	0.015	3	0.005	79.129	<0.001
	drought condition	0.013	1	0.013	205.888	<0.001
	light quality*light quality	0.005	3	0.002	27.437	<0.001

Table S2. Statistics of sample sequencing data.

Sample Name	Raw Reads (M)	Raw Base (Gb)	Clean Reads (M)	Clean Base (Gb)	Q20	Q30	GC content
0.5_1	45.62	6.84	45.57	6.81	97.33%	92.91%	43.69%
0.5_2	45.20	6.78	45.13	6.73	97.29%	92.76%	43.65%
0.5_3	41.46	6.22	41.40	6.17	96.22%	90.01%	43.76%
0_1	44.14	6.62	44.03	6.54	97.59%	93.57%	43.63%
0_2	40.28	6.04	40.21	5.99	97.64%	93.68%	43.80%
0_3	40.65	6.10	40.56	6.04	97.50%	93.34%	43.93%
1_1	39.81	5.97	39.76	5.94	96.92%	91.79%	43.96%
1_2	43.41	6.51	43.35	6.47	97.25%	92.68%	43.87%
1_3	42.90	6.44	42.86	6.41	97.31%	92.83%	43.87%
2_1	42.64	6.40	42.55	6.34	97.23%	92.49%	44.54%
2_2	41.26	6.19	41.21	6.16	97.12%	92.31%	43.88%
2_3	41.62	6.24	41.57	6.21	97.33%	92.87%	43.96%
3_1	40.76	6.11	40.71	6.09	97.23%	92.54%	44.08%
3_2	42.03	6.30	42.00	6.28	97.25%	92.54%	44.11%
3_3	43.60	6.54	43.55	6.50	96.89%	91.65%	44.11%
6_1	42.25	6.34	42.22	6.32	96.91%	91.79%	44.33%
6_2	43.87	6.58	43.81	6.54	97.05%	92.08%	46.13%
6_3	40.37	6.06	40.33	6.02	97.09%	92.22%	44.11%
D0.5_1	40.91	6.14	40.87	6.11	97.32%	92.83%	43.69%
D0.5_2	39.50	5.92	39.45	5.89	97.13%	92.34%	43.73%
D0.5_3	40.04	6.01	40.00	5.98	97.10%	92.21%	43.85%
D0_1	42.59	6.39	42.53	6.30	98.06%	94.85%	43.62%
D0_2	46.00	6.90	45.94	6.86	97.19%	92.45%	43.64%
D0_3	42.76	6.41	42.69	6.37	96.88%	91.65%	44.25%

D1_1	40.61	6.09	40.56	6.06	97.43%	93.12%	43.66%
D1_2	43.95	6.59	43.90	6.57	97.15%	92.38%	43.76%
D1_3	39.94	5.99	39.90	5.97	97.01%	92.00%	43.78%
D2_1	40.02	6.00	39.96	5.97	96.75%	91.32%	44.00%
D2_2	45.07	6.76	45.02	6.73	97.36%	92.97%	44.23%
D2_3	39.56	5.93	39.51	5.91	97.63%	93.63%	44.13%
D3_1	52.02	7.80	51.95	7.70	98.16%	95.10%	43.93%
D3_2	39.80	5.97	39.77	5.95	97.13%	92.29%	44.17%
D3_3	49.23	7.38	49.08	7.18	98.31%	95.48%	44.10%
D6_1	42.76	6.41	42.71	6.38	96.78%	91.44%	44.19%
D6_2	39.88	5.98	39.83	5.95	97.04%	92.06%	44.27%
D6_3	41.41	6.21	41.36	6.18	97.16%	92.39%	44.17%

Table S3. Reference genome alignment statistics.

	Sample	Total CleanReads	Total MappingRatio	Uniquely MappingRatio	Multi MappingRatio	Discordantly MappingRatio
Blue light without drought	0.5_1	45572362	97.20%	91.25%	3.85%	2.10%
	0.5_2	45131362	97.01%	91.28%	3.62%	2.11%
	0.5_3	41397786	96.53%	90.41%	3.58%	2.54%
	0_1	44028792	97.54%	92.25%	3.45%	1.84%
	0_2	40212418	97.33%	91.86%	3.62%	1.85%
	0_3	40560566	93.79%	88.61%	3.45%	1.73%
	1_1	39760528	96.99%	91.41%	3.24%	2.34%
	1_2	43346300	97.04%	91.32%	3.49%	2.23%
	1_3	42858950	97.29%	91.64%	3.62%	2.03%
	2_1	42554656	96.90%	90.78%	4.06%	2.06%
	2_2	41205696	97.22%	91.64%	3.18%	2.40%
	2_3	41571282	97.44%	92.23%	3.17%	2.04%
	3_1	40714442	97.44%	92.37%	3.05%	2.02%
	3_2	41999962	97.37%	92.28%	3.10%	1.99%
	3_3	43548166	97.30%	91.80%	3.20%	2.30%
	6_1	42219070	97.11%	91.82%	3.09%	2.20%
	6_2	43813732	94.50%	79.96%	11.90%	2.64%
	6_3	40326524	97.28%	92.06%	3.00%	2.22%
Blue light with drought	D0.5_1	40866116	97.33%	91.89%	3.51%	1.93%
	D0.5_2	39453888	97.20%	91.64%	3.38%	2.18%
	D0.5_3	40004238	96.31%	90.42%	3.72%	2.17%
	D0_1	42529330	97.86%	92.85%	3.61%	1.40%
	D0_2	45941578	97.33%	91.70%	3.50%	2.13%
	D0_3	42689184	93.96%	86.89%	4.94%	2.13%
	D1_1	40559058	97.30%	91.75%	3.51%	2.04%
	D1_2	43898616	97.09%	91.28%	3.64%	2.17%
	D1_3	39898858	97.21%	91.63%	3.40%	2.18%
	D2_1	39955804	96.94%	91.30%	3.19%	2.45%
	D2_2	45016632	97.28%	91.66%	3.71%	1.91%
	D2_3	39514250	97.70%	92.82%	3.21%	1.67%
	D3_1	51945964	97.98%	93.43%	3.23%	1.32%
	D3_2	39765614	97.46%	92.31%	3.09%	2.06%

Sample	Total CleanReads	Total MappingRatio	Uniquely MappingRatio	Multi MappingRatio	Discordantly MappingRatio
D3_3	49081996	97.99%	93.69%	3.16%	1.14%
D6_1	42711460	97.06%	91.78%	3.03%	2.25%
D6_2	39833872	97.26%	91.92%	3.24%	2.10%
D6_3	41358510	97.44%	92.46%	2.91%	2.07%

Table S4. The cis-acting elements of the CsPIP2;3.

Cis-acting elements	Sequence	Number	Position (Strand)	function
A-box	CCGTCC	1	1535 (-)	cis-acting regulatory element
AAGAA -motif	gGTAAAGAAA, GAAAGAA	7	216 (-), 1129 (+), 1160 (+), etc.	cis-acting element involved in gibberellin responsiveness
ABRE	ACGTG, GACACGTGGC, CACGTG, CACGTG	7	937 (-), 1390 (+), 1491 (+), etc.	cis-acting element involved in the abscisic acid responsiveness
ABRE3a	TACGTG	1	1490 (+)	cis-acting element involved in the abscisic acid responsiveness
ABRE4	CACGTA	1	1490 (-)	cis-acting element involved in the abscisic acid responsiveness
AE-box	AGAAACAA	2	213 (-),1719 (-)	part of a module for light response
ARE	AAACCA	4	24 (-), 1089 (+), 633 (-), 1516 (-)	cis-acting regulatory element essential for the anaerobic induction
AT-rich element	ATAGAAATCAA	1	1652 (+)	binding site of AT-rich DNA binding protein (ATBP-1)
AT~TATA-box	TATATA	4	762 (+), 1379 (-), 764(+), etc.	The basic site for the assembly of the RNA polymerase II transcription complex
AuxRE	TGTCTCAATAAG	1	571 (-)	part of an auxin-responsive element
Box 4	ATTAAT	2	838 (+), 846 (+)	part of a conserved DNA module involved in light responsiveness
CAAT-box	CAAAT, CCAAT, CAAT, TGCCAAC	47	18(+), 402(-), 1145(-), etc.	common cis-acting element in promoter and enhancer regions
CCGTCC motif	CCGTCC	1	1535(-)	cis-acting regulatory element
CCGTCC-box	CCGTCC	1	1535(-)	cis-acting regulatory element
ERE	ATTTTAAA	1	595 (+)	cis-acting element involved in ethylene responsiveness
G-Box	CACGTT, GCCACGTGGA, CCACGTAA, TACGTG, CACGTG,	8	937 (+), 1613(-), 1392(-), etc.	cis-acting regulatory element involved in light responsiveness

GT1-motif	GGTTAA	1	1083(-)	light responsive element
HD-Zip 1	CAAT(A/T)ATTG	1	441 (+)	element involved in differentiation of the palisade mesophyll cells
I-box	GTATAAGGCC	1	615 (+)	part of a light responsive element
MBS	CAACTG	1	1039 (+)	MYB binding site involved in drought-inducibility
MYB	CAACCA/TAACCA	4	1084 (+), 1505(-), 1155(-), etc.	MYB transcription factor binding site
MYB-like sequence	TAACCA	2	1084 (+), 1509(-)	MYB transcription factor binding site
MYC	CAATTG	4	881 (+), 1799 (-), 1097 (-)	bHLH transcription factor binding site
Myb	CAACTG	1	1039 (+)	MYB transcription factor binding site
Myc	TCTCTTA	2	898 (+), 967 (+)	bHLH transcription factor binding site
STRE	AGGGG	1	290 (+)	Stress Responsive Element
Sp1	GGGCGG	1	1085 (+)	The binding site of the general transcription factor Sp1
TATA	TATAAAAT	1	71(-)	core promoter element
TATA-box	TATAAAA, TATAAA, TATAA, TATA, TACATAAA, TAAAGATT, ATATAT, ATTATA, taTATAAAtc, TATATAA, TATATA, ATATAA, TACAAAA,	47	75 (+), 744 (+), 766 (+), etc.	The core promoter element at -30 bp from the transcription start site
TCA	TCATCTTCAT	2	328 (-), 1782 (-)	cis-acting element involved in salicylic acid responsiveness
TCA-element	CCATCTTTTT	2	555 (-), 1754 (-)	cis-acting element involved in salicylic acid responsiveness
WRE3	CCACCT	2	1426 (+), 1758 (-)	cis-acting element involved in Wound responsiveness
circadian	CAAAGATATC	1	1618 (-)	cis-acting regulatory element involved in circadian control
dOCT	CTCGGATC	1	1246(+)	The core binding sites of the transcription factor Oct family (particularly Oct-1)

Table S5. Primer sequences for qRT-PCR.

Gene ID	Primer sequence
CsPIP1;1-F	GTCCACTTGGCAACAATCCCG
CsPIP1;1-R	GATCATCCCACGCTTTGTCCT
CsPIP1;2-F	GGACTACAAGGAGCCACCAC

CsPIP1;2-R	TACTCTTGGACCGGACGACA
CsPIP1;3-F	CTGTCCCTCACAAGAGCAGTATTC
CsPIP1;3-R	TGATCTCAGCACCAAGACCG
CsPIP1;4-F	AGTCCACTTGGCAACAATTCCA
CsPIP1;4-R	ATCATCCCACGCTTTGTCCTT
CsPIP1;5-F	TGCGGTGGTCCTTTTACAGA
CsPIP1;5-R	CCAAGCAATGCCCTGAACAC
CsPIP1;6-F	TGGGTCTTCGGGGGTATGAT
CsPIP1;6-R	CAAGGCTCGGACCAACGATA
CsPIP2;1-F	TTTGGGTTGGACCTTTCATTGGA
CsPIP2;1-R	ATACTCATGGCACACAATTATTAGGCTT
CsPIP2;2-F	TTTGGGCTGTTTTTGGCTCG
CsPIP2;2-R	CACCGCCGTAACGAGTGTAA
CsPIP2;3-F	AGCGGTGACTTTTGGGTTGT
CsPIP2;3-R	CCACCTCCGTGCTCATTGTA
CsPIP2;4-F	TCTCCGGTGGCCACATTAAC
CsPIP2;4-R	CAAGCCACAGCCGCAAATAG
CsPIP2;5-F	TGGCGAAGGACTACCAGGAT
CsPIP2;5-R	TCCCCATACCATGCGTTTCC
CsPIP2;6-F	TGCCACGCTTCTCTTCTTGT
CsPIP2;6-R	CAGGAAATGCCTAAAGCGCC
CsPIP2;7-F	CGTAAAGTGTCGTTGGTGCG
CsPIP2;7-R	AACCGGTGCCTTTGGAGTAG
CsPIP2;8-F	AAAGAAGTCACGGAAGAGGGAC
CsPIP2;8-R	AAAGATCATGCCACCGAAGG
pSuper1300-eGFP-CsPIP2;3-F	ATCGACTCTAGAAAGCTTATGTCTAAGGATCTTGAAGCTGGT
pSuper1300-eGFP-CsPIP2;3-R	GGTACCGGATCCACTAGTTACGGCAGTCGAACTTCTGAA
CsPIP12pro-pBI121-F	TATGACCATGATTACGCCAAGCTTATTACTTCTCCCAACATGGATACAA
CsPIP12pro-pBI121-R	AAGGGACTGACCACCCGGGGATCCTCCGGAAAAACGTGTGTGTT
CsPIP23pro-pBI121-F	TATGACCATGATTACGCCAAGCTTTGATCAGGTATGGATTTGAAGAAAATAGT
CsPIP23pro-pBI121-R	AAGGGACTGACCACCCGGGGATCCGGCTTGTGTTGGTACTTGG
